# Anti-TNF alpha-induced paradoxical psoriasis treated with topical roflumilast 0.3%

**DOI:** 10.1016/j.jdcr.2024.03.015

**Published:** 2024-04-02

**Authors:** Edith Hanna, Anissa Benchikh El Fegoun

**Affiliations:** a Department of Dermatology, Centre Hospitalier Régional du Grand-Portage, CISSS du Bas-St-Laurent, Rivière-du-Loup, Québec, Canada; b Faculty of Medicine, Université Laval, Québec City, Québec, Canada

**Keywords:** Anti-TNF, Anti-TNF alpha, paradoxical psoriasis, paradoxical psoriasis treated with topical roflumilast 0.3%, PDE-4 inhibitor, PDE-4 treatment for paradoxical psoriasis, psoriasis, roflumilast, roflumilast 0.3%

## Introduction

Anti-TNF alpha treatments have revolutionized the treatment for chronic inflammatory diseases such as rheumatoid arthritis, inflammatory colitis, Crohn's disease, ulcerative colitis, and psoriasis vulgaris.^1^ However, there have been a number of adverse effects associated with such treatments, mainly paradoxical psoriasis which is of particular importance to dermatologists. Some patients are more at risk, such as those carrying the IL 23-R SNIP gene.^1^ The delay of onset for paradoxical psoriasis is variable, ranging from 1 to 30 months after introduction of anti-TNF alpha injections. Treatment options for paradoxical psoriasis remain limited, prompting either the addition of topical agents to minimize cutaneous inflammation or the eventual discontinuation of anti-TNF alpha injections.^2^ Roflumilast cream 0.3% is a highly selective and potent topical phosphodiesterase-4 (PDE-4) inhibitor approved in 2022 by the Food and Drug Administration (FDA) for the treatment of psoriasis, including intertriginous areas. We report a case of a 61-year-old male with new-onset paradoxical palmoplantar psoriasis after adalimumab initiation that was successfully treated with roflumilast 0.3% once daily cream.

## Case report

**Fig 1:**
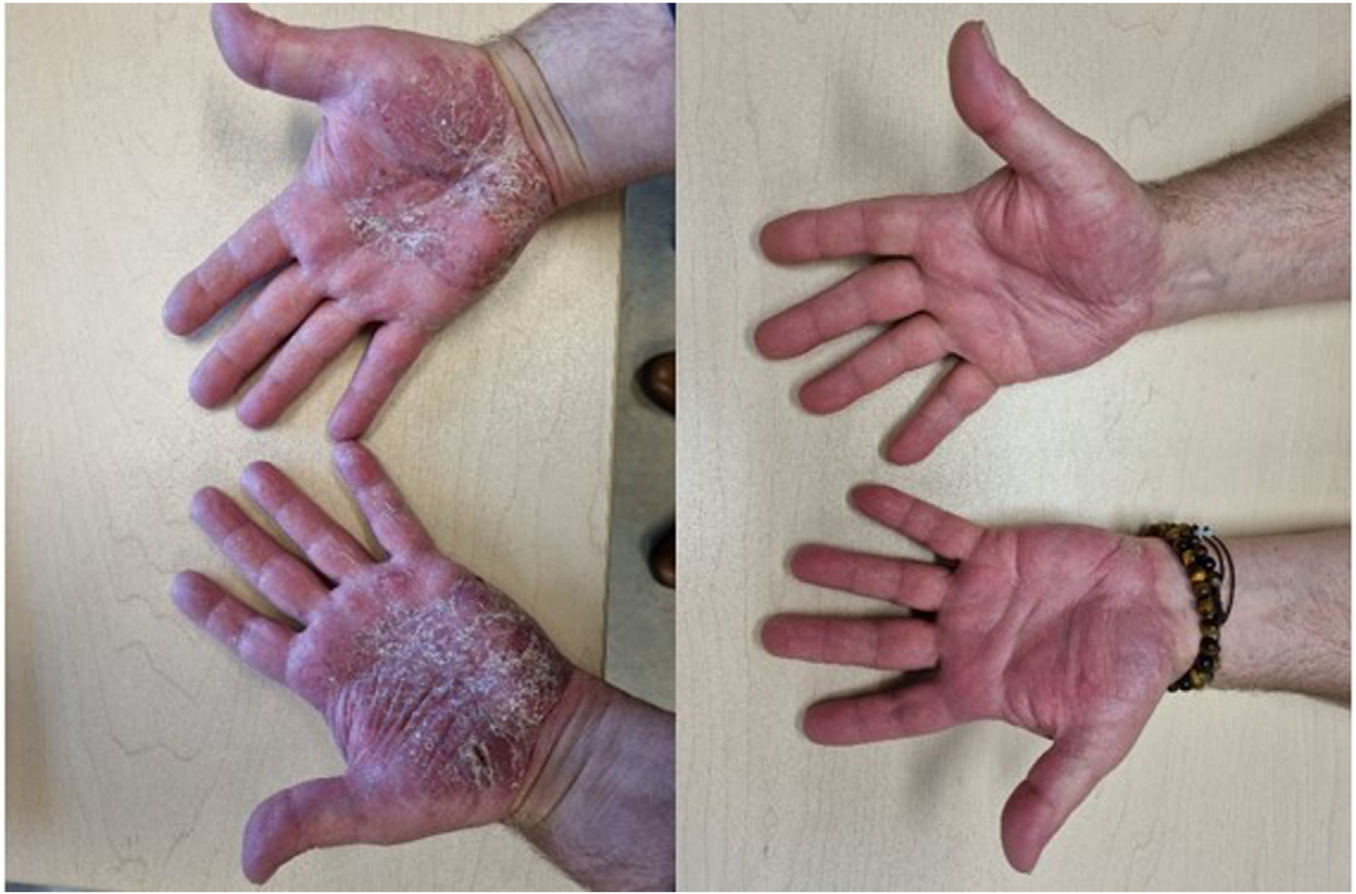
Before (*left* photo) and after (*right* photo) 1 month initiation of topical roflumilast 0.3% cream once daily.

We report the case of a 61-year-old man with a history of dyslipidemia, duodenitis, and colonic adenoma, being followed for stage 3 hidradenitis suppurativa (HS) since December 2022. The patient was started on adalimumab in April of 2023 which resulted in 80% improvement of his HS. In July of 2023, the patient presented with a painful and itchy eruption over the palms and legs. This was approximately 4 months after starting adalimumab. No other inciting event was identified prior to the development of his rash. Upon examination, the patient had palmoplantar pustulosis along with psoriasis plaques on his shins consistent with a diagnosis of paradoxical psoriasis, an adverse reaction to his TNF inhibitor. Adalimumab was immediately discontinued. He was initially treated with clobetasol cream twice daily for 1 month and was switched to guselkumab 1 month later. His paradoxical psoriasis deteriorated with the appearance of new psoriasis plaques on his right forearm. Treatment was modified thereafter to calcipotriol and betamethasone dipropionate (daivobet) gel once daily for a month. After 6 weeks, the patient noted that daivobet gel was causing a burning sensation on his hands leading to difficulty forming a fist. He added that his HS reactivated with new nodules appearing in his right underarm and right inguinal area. It was therefore, decided to discontinue guselkumab and initiate bimekizumab which could address both his psoriasis and HS. Roflumilast cream 0.3% once daily was prescribed in the meantime until his biologic would get approved. Subsequent follow-up 1 month later showed complete resolution in his palmoplantar psoriasis after exclusive use of roflumilast cream 0.3% once daily (Fig 1). He is still awaiting the initiation of bimekizumab.

## Discussion

Paradoxical psoriasis is an inflammatory dermatosis on the borderline with an unclear pathogenesis, occurring in 1% to 5% of individuals using anti-TNF alpha drugs. It is highly frequent, seen with all anti-TNF alpha molecules, with a risk of recurrence of over 50%.^2^ TNF alpha is a powerful pro-inflammatory cytokine which coordinates immune responses and plays an important role in limiting the spread of infectious pathogens. Anti-TNF alpha agents can lead to an increased risk of infections, such as the reactivation of latent tuberculosis or hepatitis B. Paradoxically, anti-TNF alpha treatments can also aggravate pre-existing autoimmune diseases or induce new inflammatory diseases, such as lupus erythematosus and paradoxical psoriasis.^3^ In paradoxical psoriasis, there is no negative feedback leading to continuous IFNα overproduction which will block the activation of T-cell autoimmunity.^4^

The greatest challenge today is its management. In milder cases of paradoxical psoriasis, anti-TNF alpha therapy can often be continued, and the same treatments can be utilized as for psoriasis vulgaris: topical corticosteroids, vitamin D analogues, keratolytics, phototherapy or even systemic treatments such as methotrexate and cyclosporin.^5^ For moderate to severe cases of paradoxical psoriasis, or those resistant to conventional treatment, the anti-TNF alpha therapy is typically switched to another anti-TNF alpha molecule. If, despite the change, the lesions persist, anti-TNF alpha therapy may need to be discontinued while introducing another biologic agent such as an IL-12/IL-23 inhibitor or an IL-23 inhibitor, demonstrating the challenge of managing paradoxical psoriasis.^5^ In our patient's case, roflumilast cream 0.3% once daily could represent an alternative topical treatment for palmoplantar paradoxical psoriasis and further research is warranted to better understand the benefit and risk in the treatment of this disease.

## Conflicts of interest

None disclosed.

## References

[1] Nidegger A., Mylonas A., Conrad C. (2019). Psoriasis paradoxal induit par anti-TNF un challenge en clinique. Rev Med Suisse.

[2] Deker M. (2022). https://www.dermatologie-pratique.com/journal/article/0010055-psoriasis-tous-ses-etats.

[3] Paul C., Viguier M., Villani A. (2020). Impact des recommandations françaises sur les habitudes de prescription des traitements systémiques pour le psoriasis modéré à sévère [Les formes de psoriasis autres que le psoriasis en plaques : prise en charge au quotidien]. Les Journées Dermatologiques de Paris.

[4] Said El Mabrouk R. (2022). Psoriasis paradoxal induit par la biothérapie : à propos de 4 cas. Rev Med Interne.

[5] Harrison M.J., Dixon W.G., Watson K.D. (2009). Rates of new-onset psoriasis in patients with rheumatoid arthritis receiving anti-tumour necrosis factor alpha therapy: results from the British Society for Rheumatology Biologics Register. Ann Rheum Dis.

